# Plantar fascial fibromatosis and herpes zoster

**DOI:** 10.1371/journal.pone.0259942

**Published:** 2021-11-24

**Authors:** Chao-Yu Hsu, Der-Shin Ke, Cheng-Li Lin, Chia-Hung Kao

**Affiliations:** 1 Department of Medical Education, Ditmanson Medical Foundation, Chia-Yi Christian Hospital, Chia-Yi, Taiwan; 2 Department of Optometry/Medical Imaging and Radiological Sciences, Central Taiwan University of Science and Technology, Taichung, Taiwan; 3 Center for General Education, National Taichung University of Science and Technology, Taichung, Taiwan; 4 Department of General Education, National Chin-Yi University of Technology, Taichung, Taiwan; 5 Rural Generalist Program Japan, GENEPRO, Asahi Shi, Japan; 6 Management Office for Health Data, China Medical University Hospital, Taichung, Taiwan; 7 College of Medicine, China Medical University, Taichung, Taiwan; 8 Graduate Institute of Biomedical Sciences, College of Medicine, China Medical University, Taichung, Taiwan; 9 Department of Nuclear Medicine and PET Center, China Medical University Hospital, Taichung, Taiwan; 10 Department of Bioinformatics and Medical Engineering, Asia University, Taichung, Taiwan; 11 Center of Augmented Intelligence in Healthcare, China Medical University Hospital, Taichung, Taiwan; Hualien Tzu Chi Hospital Buddhist Tzu Chi Medical Foundation, TAIWAN

## Abstract

**Purpose:**

Infection, chronic pain and depression are considered risk factors for herpes zoster (HZ). However, the correlation between plantar fascial fibromatosis (PFF) and HZ remains unknown. This study investigated HZ risk in patients with PFF.

**Methods:**

Data was extracted from the Longitudinal Health Insurance Database 2000, which is a subsample of the Taiwan National Health Insurance (NHI) Research Database and contains 1 million NHI beneficiaries. Between 2000 and 2012, patients diagnosed as having PFF were included in the case cohort. Every case was age and sex-matched with individuals without PFF through 1:4 frequency matching (control cohort). The end of the follow-up was defined as December 31, 2013, the date of HZ diagnosis, death, emigration, or withdrawal from the NHI program.

**Results:**

In total, 4,729 patients were diagnosed as having PFF and were matched with 18,916 individuals without PFF. Patients with PFF were 1.23 times more likely to develop HZ than were those without PFF. Among those aged ≥65 years, patients with PFF had a higher HZ risk than did those without PFF (adjusted hazard ratio [aHR] = 1.48). Men with PFF had a significantly higher risk of HZ than did men without PFF (aHR = 1.44).

**Conclusion:**

Patients with PFF, particularly older and male patients, having a high HZ risk and may thus be vaccinated for HZ.

## Introduction

Plantar fascial fibromatosis (PFF), also known as Ledderhose’s disease, was first described by Ledderhose in 1897 [1]. PFF is characterized by a benign, slow-growing nodule forming in the plantar fascia. Over time, nodule growth may cause walking to become painful.

PFF prevalence is poorly understood but is most commonly seen in young adults. The previous studies have shown that the prevalence of PFF in men is twice that of women. PFF presents bilaterally in 25% of patients [2–4]. Although PFF can be diagnosed through physical examination alone, ultrasound or magnetic resonance imaging can be used to rule out other diseases and confirm PFF. Conservative treatment modalities, including steroid injection, radiation, and extracorporeal shock wave therapy, and surgical intervention are used to treat PFF [3, 4].

Herpes zoster (HZ) is characterized by painful vesicular skin rashes in affected areas caused by the reactivation of the varicella-zoster virus from its latent state in posterior dorsal root ganglions. A systematic review of the literature determined that the incidence rate of HZ in the general population was between 2.1 and 5.5 per 1,000 person-years. The HZ incidence rate was higher in patients with underlying conditions such as diabetes (9.4–15.3 per 1000 person-years) or chronic obstructive pulmonary disease (11.0–11.4 per 1000 person-years). The highest HZ incidence rate (up to 400.0 per 1,000 person-years) was observed in immunocompromised patients [5].

HZ incidence was reported to increase with age because of the age-related attenuation of immunity. The HZ incidence rate was high in adults aged 75–79 years (9.12 per 1,000 person-years) [6]. The 10-year recurrence risk of HZ was 10.26% [7]. Postherpetic neuralgia is an unpleasant complication that can last from months to years after recovery from the acute stage.

The burden of diseases such as infection [8, 9], chronic pain related diseases [10–14] and depression [15] has been considered to cause stress in affected patients and increase HZ risk. Both PFF and PFF-related pain may also cause stress in affected individuals and thus increase HZ risk. In this study, we investigated the association between PFF and HZ risk.

## Materials and methods

### Patients

A unique (single-payer) program was operated by the Taiwan National Health Insurance (NHI) since March 1, 1995. Approximately 99.9% of Taiwanese residents are enrolled. In this study, data was extracted from the Longitudinal Health Insurance Database 2000 (LHID2000) which was a subsample of the NHI Research Database (NHIRD) containing 1 million NHI beneficiaries. A de-identification process was applied before analysis to ensure the patients’ privacy. The International Classification of Diseases, Ninth Revision, Clinical Modification (ICD-9-CM) was used to identify diagnoses.

### Study population

Patients with PFF (ICD-9-CM: 728.71) were assigned to the case cohort. The index date for the PFF group was the date of the first PFF diagnosis. Patients aged ≤20 years or those who were diagnosed as having as HZ (ICD-9-CM: 053) before the index date were excluded. A total of 4,729 patients were diagnosed as having PFF between 2000 and 2012; they were age and sex-matched with 18,916 individuals not diagnosed as having PFF between 2000 and 2012 through 1:4 frequency matching. The reference date for the non-PFF group was set as the index date of their age and sex-matched PFF counterpart. The end of the follow-up period was defined as either the end of 2013 or the date on which individuals were diagnosed as having HZ, died, emigrated or withdrew from the NHI program. Some comorbidities correlated to HZ were selected, namely diabetes (ICD-9-CM: 250), coronary artery disease (CAD; ICD-9-CM: 410–414), depression (ICD-9-CM: 296.2, 296.3, 300.4, and 311), chronic kidney disease (ICD-9-CM: 585 and 586), obesity (ICD-9-CM: 278), and cancer (ICD-9-CM: 140–208). Postherpetic neuralgia is the most common complication after recovery from HZ. In this paper, postherpetic neuralgia and recurrence of HZ were also analyzed.

### Ethics approval and consent to participate

The NHIRD encrypts patient personal information to protect privacy and provides researchers with anonymous identification numbers associated with relevant claims information, including sex, date of birth, medical services received, and prescriptions. Therefore, patient consent is not required to access the NHIRD. This study was approved to fulfill the condition for exemption by the Institutional Review Board (IRB) of China Medical University (CMUH104-REC2-115-AR4). The IRB also specifically waived the consent requirement.

### Statistical analysis

To explore the association between PFF and HZ, the incidence of HZ in patients with and individuals without PFF was calculated, and the risk of HZ was evaluated using the Cox proportional hazards regression. Hazard ratios with 95% confidence intervals (95% CI) were calculated to estimate the risk, and adjusted hazard ratios (aHR) with 95% CI were also calculated after adjusting for statistically significant confounding factors in the models. The Kaplan-Meier curves of the cumulative HZ incidence in patients with and individuals without PFF were used to depict the difference between the cohorts, and the log-rank test was also performed.

Categorical data was presented as counts and percentages; the chi-square test was used to examine the differences between demographic distributions and the comorbidities of the case and control cohorts. Continuous data was presented as means and standard deviations (SD), and the *t*-test was used to compare mean values between the case and control cohorts for each continuous variable. Statistical analysis was performed using SAS 9.4 (SAS Institute Inc., Cary, NC). The plots were created using R language. A *p* of ≤0.05 was considered statistically significant.

## Results

### Baseline characteristics

The distribution of demographic characteristics and comorbidities in the cohorts with and without PFF was listed in the Table 1. Both cohorts, 41.0%, 41.7% and 17.3% of the individuals were aged ≤49, 50–64, and ≥65 years, respectively, with a mean (± SD) age of 52.3 (± 13.1) and 51.9 (± 13.5) years in the case and control cohorts, respectively. Moreover, 63.2% were women and 36.8% were men. Both the cohorts did not exhibit significant differences in investigated comorbidities, namely diabetes, chronic kidney disease, and cancer.

**Table 1:** Demographic characteristics and comorbidities in cohorts with and without plantar fascial fibromatosis.

	Plantar facial fibromatosis		
	No	Yes	
Variable	N = 18916	N = 4729	*p*-value
**Age, year**			0.99
≤ 49	7752(41.0)	1938(41.0)	
50–64	7892(41.7)	1978(41.7)	
65+	3272(17.3)	818(17.3)	
Mean±SD^†^	51.9±13.5	52.3±13.2	<0.001
**Sex**			0.99
Female	11960(63.2)	2990(63.2)	
Male	6956(36.8)	1739(36.8)	
**Comorbidity**			
Diabetes	1231(6.51)	307(6.49)	0.97
CAD	2599(13.7)	842(17.8)	<0.001
Depression	1056(5.58)	321(6.79)	0.002
Chronic kidney disease	307(1.62)	63(1.33)	0.15
Obesity	311(1.64)	203(4.29)	<0.001
Cancer	557(2.94)	135(2.85)	0.74

Chi-Square Test;

^†^: T-Test

CAD denotes coronary artery disease

### Association of risk factors with HZ and Kaplan-Meier plot in the cohorts

**Fig 1:**
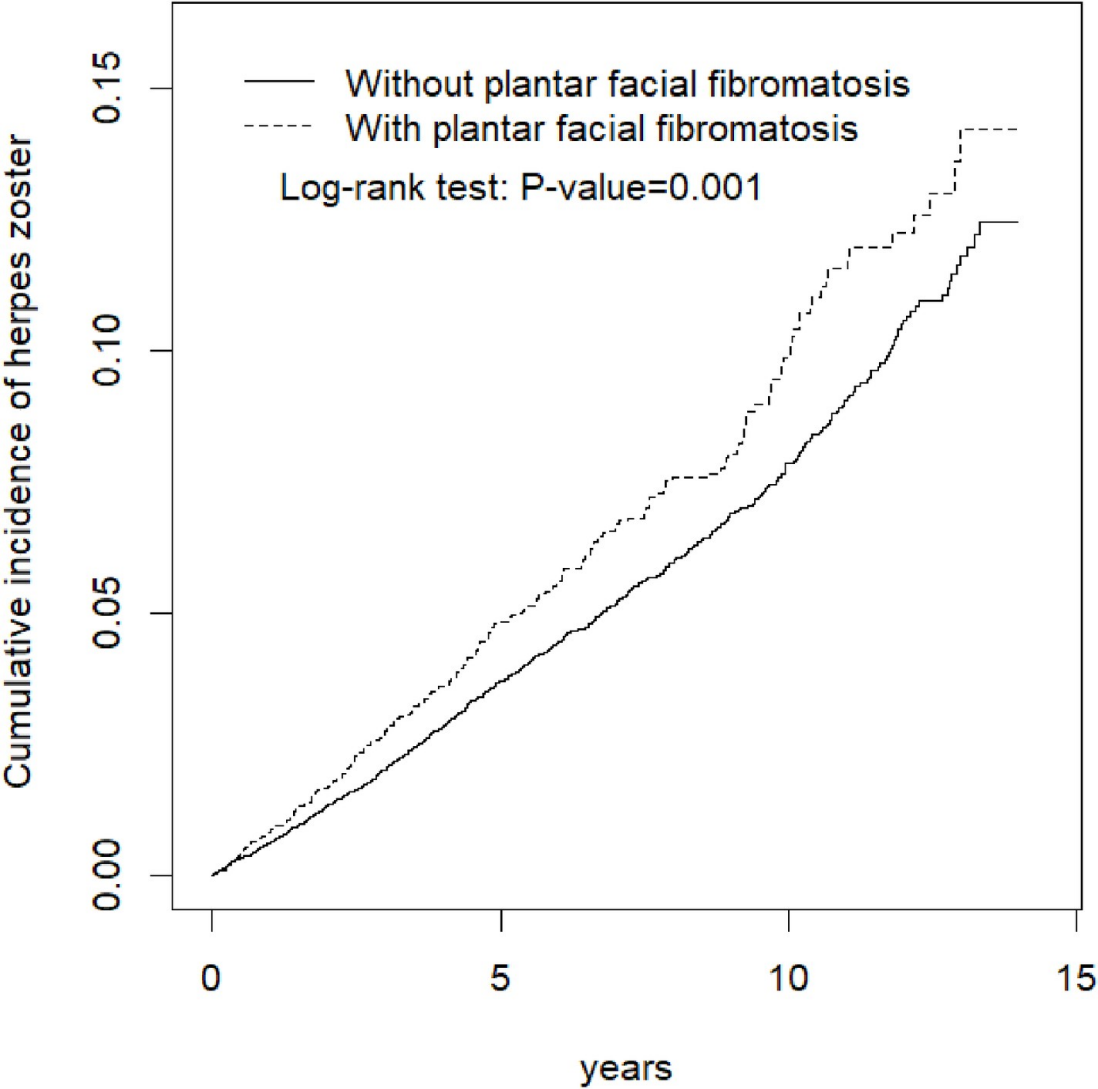
Comparison of cumulative incidence of herpes zoster for patients with (dashed line) and without (solid line) plantar fascial fibromatosis.

PFF, age, sex, and comorbidities (diabetes, CAD, depression, chronic kidney disease, and cancer) were determined to be significant risk factors for HZ (Table 2). Patients with PFF were 1.23 times more likely to develop HZ than were those without PFF after adjustments for age, sex, and comorbidities (Fig 1).

**Table 2:** The incidence and risk factors for herpes zoster.

Variables	Event	PY	Rate^#^ (95% CI)	Crude HR (95% CI)	Adjusted HR^&^ (95% CI)
**Plantar fascial fibromatosis**					
No	834	105562	7.90(7.60, 8.23)	1.00	1.00
Yes	265	26587	9.97(9.28, 10.8)	1.26(1.10, 1.45)**	1.23(1.07, 1.41)**
**Age, year**					
≤ 49	250	56409	4.43(4.17, 4.70)	1.00	1.00
50–64	566	54171	10.5(9.95, 11.0)	2.38(2.05, 2.76)***	2.21(1.89, 2.57)***
65+	283	21568	13.1(12.2, 14.3)	3.01(2.54, 3.57)***	2.65(2.21, 3.18)***
**Sex**					
Female	788	85062	9.26(8.92, 9.66)	1.40(1.23, 1.60)***	1.26(1.10, 1.44)***
Male	311	47087	6.60(6.22, 7.01)	1.00	1.00
**Comorbidities**					
** Diabetes**					
No	1000	124051	8.06(7.75, 8.40)	1.00	1.00
Yes	99	8097	12.2(10.7, 14.0)	1.54(1.25, 1.89)***	1.07(0.87, 1.33)
** CAD**					
No	857	113781	7.53(7.23, 7.83)	1.00	1.00
Yes	242	18368	13.2(12.0, 14.0)	1.77(1.53, 2.04)***	1.25(1.07, 1.46)**
** Depression**					
No	1026	125644	8.17(7.91, 8.48)	1.00	1.00
Yes	73	6505	11.2(9.75, 12.9)	1.43(1.13, 1.82)**	1.18(0.93, 1.51)
** Chronic kidney disease**					
No	1076	130543	8.24(7.99, 8.57)	1.00	1.00
Yes	23	1605	14.3(11.0, 18.7)	1.80(1.19, 2.73)**	1.31(0.86, 1.99)
** Obesity**					
No	1083	129722	8.35(8.07, 8.65)	1.00	1.00
Yes	16	2427	6.59(5.14, 8.48)	0.82(0.50, 1.35)	
** Cancer**					
No	1062	129118	8.23(7.91, 8.48)	1.00	1.00
Yes	37	3030	12.2(10.1, 14.8)	1.54(1.11, 2.14)*	1.18(0.85, 1.65)

Rate^#^, incidence rate, per 1,000 person-years; Crude HR, relative hazard ratio; Adjusted HR^&^, multivariable analysis including age, sex, and comorbidities of diabetes, CAD, depression, chronic kidney disease, and cancer;

*p<0.05,

**p<0.01,

***p<0.001

### Stratified analysis of the association between PFF and HZ

Patients with PFF aged ≥65 years had a higher HZ risk than did their non-PFF counterparts (aHR = 1.48, 95% CI = 1.14–1.92). Men with PFF had a significantly higher HZ risk than did those without PFF (aHR = 1.44, 95% CI = 1.12–1.85). Patients with PFF and any comorbidity had a higher HZ risk than did those without PFF (aHR = 1.35, 95% CI = 1.09–1.69) (Table 3).

**Table 3:** Incidence of herpes zoster by age, sex and comorbidity and Cox model measured hazards ratio for patients with plantar facial fibromatosis compared to those without plantar fascial fibromatosis.

	Plantar fascial fibromatosis							
	No			Yes				
Variables	Event	PY	Rate^#^ (95% CI)	Event	PY	Rate^#^ (95% CI)	Crude HR (95% CI)	Adjusted HR^&^ (95% CI)
**Age, years**								
≤ 49	187	45109	4.15(3.89,4.43)	63	11300	5.58(4.89,6.35)	1.34(1.01, 1.79)*	1.31(0.98, 1.75)
50–64	443	43339	10.2(9.66,10.9)	123	10832	11.4(10.2,12.8)	1.11(0.91, 1.36)	1.10(0.90, 1.34)
65+	204	17113	11.9(10.9,13.0)	79	4454	17.7(15.0,21.1)	1.48(1.14, 1.92)**	1.48(1.14, 1.92)**
**Sex**								
Female	609	68002	8.96(8.57,9.37)	179	17060	10.5(9.56,11.6)	1.17(0.99, 1.38)	1.15(0.98, 1.36)
Male	225	37560	5.99(5.57,6.41)	86	9527	9.03(7.99,10.3)	1.51(1.18, 1.93)**	1.44(1.12, 1.85)**
**Comorbidities** ^§^								
No	570	82086	6.94(6.60,7.30)	149	18627	8.00(7.30,8.83)	1.15(0.96, 1.38)	1.13(0.95, 1.36)
Yes	264	23476	11.3(10.5,12.2)	116	7960	14.6(12.8,16.6)	1.29(1.04, 1.61)*	1.35(1.09, 1.69)**

Rate^#^, incidence rate, per 1,000 person-years; Crude HR, relative hazard ratio; Adjusted HR^&^, multivariable analysis including age, sex, and comorbidities of diabetes, CAD, depression, and chronic kidney disease;

^§^: Individuals with any comorbidity of diabetes, CAD, depression, and chronic kidney disease, obesity, and cancer were classified into the comorbidity group;

*p<0.05,

**p<0.01

The joint effects of comorbidities and PFF on HZ risk was illustrated in Table 4. The statistically significant higher risk of HZ was observed in the patients with both PPF and CAD (aHR = 1.63, 95% CI = 1.28–2.08) than those without PPF and CAD. Postherpetic neuralgia was no difference between the patients with and without PFF (15.1% vs 10.4%) (adjusted odds ratio [aOR] = 1.21 (95% CI = 0.89–1.64) (Table 5). However, recurrence of HZ was slight difference but statistically significant between the patients with and without PFF (32.1% vs 27.9%) (aOR = 1.51 (95% CI = 1.00–2.27).”

**Table 4:** Cox method estimated hazard ratios of herpes zoster associated plantar fascial fibromatosis and comorbidities.

Variables		N	Event (n)	Adjusted HR^†^ (95% CI)	p-value^#^
Plantar fascial fibromatosis	Diabetes				0.81
No	No	17685	760	1(Reference)	
No	Yes	1231	74	1.09(0.85, 1.38)	
Yes	No	4422	240	1.24(1.07, 1.43)**	
Yes	Yes	307	25	1.38(0.93, 2.06)	
Plantar fascial fibromatosis	CAD				0.32
No	No	16317	669	1(Reference)	
No	Yes	2599	165	1.12(0.94, 1.34)	
Yes	No	3887	188	1.16(0.99, 1.37)	
Yes	Yes	842	77	1.63(1.28, 2.08)***	
Plantar fascial fibromatosis	Depression				0.50
No	No	17860	780	1(Reference)	
No	Yes	1056	54	1.25(0.95, 1.65)	
Yes	No	4408	246	1.24(1.08, 1.44)**	
Yes	Yes	321	19	1.46(0.92, 2.30)	
Plantar fascial fibromatosis	Chronic kidney disease				0.83
No	No	18609	816	1(Reference)	
No	Yes	307	18	1.32(0.83, 2.12)	
Yes	No	4666	260	1.24(1.08, 1.43)**	
Yes	Yes	63	5	1.47(0.61, 3.55)	
Plantar fascial fibromatosis	Obesity				
No	No	18605	826	1(Reference)	0.56
No	Yes	311	8	0.71(0.35, 1.42)	
Yes	No	4526	257	1.24(1.08, 1.43)**	
Yes	Yes	203	8	1.15(0.57, 2.30)	
Plantar fascial fibromatosis	Cancer				0.69
No	No	18359	805	1(Reference)	
No	Yes	557	29	1.21(0.83, 1.75)	
Yes	No	4594	257	1.25(1.09, 1.44)**	
Yes	Yes	135	8	1.26(0.63, 2.53)	

^†^ Model was adjusted for age and sex;

^#^p-value for interaction;

**p<0.01;

***p < 0.001

**Table 5:** Postherpetic neuralgia and recurrence of herpes zoster, and estimated odds ratio by logistic regression analysis.

	Plantar fascial fibromatosis	
	No	Yes
	n/N	n/N
**Postherpetic neuralgia**	87/834	40/265
Rate, %	10.4	15.1
cOR (95% CI)	1 (Reference)	1.22(0.90, 1.64)
aORs (95% CI) ^a^	1 (Reference)	1.21(0.89, 1.64)
**Recurrence of HZ**	233/834	85/265
Rate, %	27.9	32.1
cOR (95% CI)	1 (Reference)	1.53(1.02, 2.28)*
aORs (95% CI) ^a^	1 (Reference)	1.51(1.00, 2.27)*

^a^Adjusted for age, sex, and comorbidities of diabetes, CAD, depression, and chronic kidney disease

Abbreviations: cOR, crude odds ratio; aOR, adjusted odds ratio

*p<0.05

### Sensitivity analysis

We used logistic regression to calculate the propensity score for PFF status by estimating the assignment probability based on baseline variables, including age, sex, comorbidities of diabetes, CAD, depression, chronic kidney disease, obesity, and cancer. The HZ risk was higher in PFF patients than in propensity score-matched non-PFF patients (aHR = 1.35, 95% CI = 1.12–1.62).

## Discussion

This is the first population-based study to identify the association between PFF and HZ; patients with PFF were 1.23 times more likely to develop HZ than were those without PFF.

In this study, PFF prevalence was found to be 0.5% (Table 1). Furthermore, the majority (82.7%) of patients with PFF were aged <65 years. Carroll et al. reported that PFF was most commonly seen in patients aged 20–40 years [3]. Heyd et al. reported that the onset of PFF symptoms was observed in patients aged 30–40 years [2]. However, we determined that the prevalence was similar in patients aged ≤49 (41.0%) and 50–65 (41.7%) years. PFF has a multifactorial etiology such as traumatic causes, Dupuytren’s contractures, diabetes mellitus or alcohol consumption [3, 4]. The etiology of men having a higher prevalence than women is still unclear [3, 4]. In contrast to previous studies [2–4], we observed that women were affected nearly 1.7 times as often as men (63.2% vs. 36.8%). This may due to the difference of tolerance for pain lead to a higher rate of women seeking medical treatment. Moreover, the data for this study was extracted from the LHID2000 which had a large sample size with 1 million insured persons. Thus, the results can be trustable. Despite Although the prevalence of PFF was higher in women in the present study, but we observed that the burden of PFF for HZ development was higher in men. Men with PFF were 1.44 times more likely to develop HZ than were men without PFF. Therefore, PFF may be a larger source of stress in men.

Although PFF is a benign disease, it can become a painful condition during walking as the nodules grow. Several studies have reported that chronic pain is associated with depression. Rapti et al. reported that 22.5% of patients with chronic pain had depression, the majority of whom (62%) were women [16]. A large sample study from the Healthcare Cost and Utilization Project database in the United States conducted by Orhurhu et al. reported that 22.9% of patients with chronic pain were diagnosed as having depression. The authors investigated the trends of depression among patients with chronic pain, and reported that the rates of depression were 22.6% in 2011 and 23.1% in 2015 [17]. Among elderly patients with chronic pain, the prevalence of depression was higher. Morete et al. reported that 35.2% of elderly patients with chronic pain were diagnosed as having depression [18]. Orhurhu et al. noted a significant increasing trend of depression among patients with chronic pain aged 65–84 years, and the rates were 29.0% in 2011 and 32.4% in 2015 [17]. Humo et al. attempted to explain the association between chronic pain and depression through molecular mechanisms. They suggested that the polymorphisms of the 5-hydroxytryptamine transporter and an inhibitory or excitatory imbalance of neurotransmission may be the reasons [19].

The association between depression and HZ has been reported by two population-based studies [15, 20]. Choi et al. reported that HZ prevalence in patients with depression was significantly higher than that in patients without depression (6.8% vs. 6.3%) [20], and Liao et al. reported an HZ incidence of 4.58 per 1,000 person-years in patients with depression, but that of only 3.54 per 1,000 person-years in those without depression. The incidence of HZ was 1.3-fold higher in patients with depression than in those without depression [15]. Both studies have reported that patients with depression had a higher risk to develop HZ. Furthermore, both studies have indicated that HZ risk was higher in middle-aged patients with depression [15, 20]. Zorrilla et al. found that depression was associated with reduced lymphocyte proliferative response and a reduction of T-cell proportion [21]. Miller found that T cells played a role in depression through a downregulation of inflammatory response. T cells might induce neuroprotective and anti-inflammatory effects during stress and inflammation, a damaged T cell function might result to the occurrence of depression [22]. Because varicella-zoster virus specific cellular immunity markedly declined in depressed patients, the infection rate of HZ was higher [23].

Livengood et al. described that physical or psychologic stress stimulates neural, hormonal, and behavioral activity designed to restore homeostasis. They considered that both pain and stress cause changes in the perceptual and stress systems, resulting in the abnormal output patterns of the body’s neuromatrix [24]. PFF and PFF-related syndromes, such as pain and depression are believed to be powerful stressors. Therefore, HZ risk among patients with PFF is high.

This retrospective study was performed using Taiwan’s NHIRD. However, this study has several limitations. First, bias when diagnosing (either PFF or HZ) may exist between specialists and general practitioners. However, all diagnoses and results in the NHIRD are verified by the NHI Administration, which is operated by the Taiwanese government, and all insurance claims were reviewed by medical specialists. Therefore, the diagnosis codes are reliable. Second, data on the severity of the disease were not available in the NHIRD. The severity of a disease may influence the decision making regarding treatment and prognosis. Third, data on lifestyle were also not available. Lifestyle, such as diet or exercise, may influence the immunity of the human body. Smoking may also influence the occurrence of HZ [25]. Although there are several limitations, using population-based data can avoid selection bias, and provide powerful statistical outcomes.

## Conclusion

Compared with individuals without PFF, patients with PFF, particularly those older and male patients, had a higher HZ risk. Vaccination against HZ may thus be essential for patients with PFF.

## Supporting information

S1 File (PDF)

S1 Checklist The RECORD statement–checklist of items, extended from the STROBE statement, that should be reported in observational studies using routinely collected health data. (DOCX)

## Abbreviations

HZ: herpes zoster
PFF: plantar fascial fibromatosis
NHI: National Health Insurance
aHR: adjusted hazard ratio
LHID2000: Longitudinal Health Insurance Database 2000
ICD-9-CM: International Classification of Diseases, Ninth revision, Clinical Modification

## References

[1] Dürr HR, Krödel A, Trouillier H, Lienemann A, Refior HJ. Fibromatosis of the plantar fascia: diagnosis and indications for surgical treatment. Foot Ankle Int. 1999; 20: 13–17. doi: 10.1177/107110079902000103 9921766

[2] Heyd R, Dorn AP, Herkströter M, Rödel C, Müller-Schimpfle M, Fraunholz I. Radiation therapy for early stages of morbus Ledderhose. Strahlenther Onkol. 2010; 186: 24–29. doi: 10.1007/s00066-009-2049-x 20082184

[3] Carroll P, Henshaw RM, Garwood C, Raspovic K, Kumar D. Plantar fibromatosis: pathophysiology, surgical and nonsurgical therapies: an evidence-based review. Foot Ankle Spec. 2018; 11: 168–176. doi: 10.1177/1938640017751184 29310463

[4] Young JR, Sternbach S, Willinger M, Hutchinson ID, Rosenbaum AJ. The etiology, evaluation, and management of plantar fibromatosis. Orthop Res Rev. 2019; 11: 1–7. doi: 10.2147/ORR.S154289 30774465 PMC6367723

[5] Mareque M, Oyagüez I, Morano R, Casado MA. Systematic review of the evidence on the epidemiology of herpes zoster: incidence in the general population and specific subpopulations in Spain. Public Health. 2019; 167: 136–146. pmis: doi: 10.1016/j.puhe.2018.10.015 30660981

[6] Salvetti A, Ferrari V, Garofalo R, Gazzaniga P, Guerroni A, Metrucci A, et al. Incidence of herpes zoster and postherpetic neuralgia in Italian adults aged ≥50 years: a prospective study. Prev Med Rep. 2019; 14: 100882. doi: 10.1016/j.pmedr.2019.100882 31193254 PMC6522697

[7] Tseng HF, Bruxvoort K, Ackerson B, Luo Y, Tanenbaum H, Tian Y, et al. The epidemiology of herpes zoster in immunocompetent, unvaccinated adults ≥50 years old: incidence, complications, hospitalization, mortality, and recurrence. J Infect Dis. 2020; 222(5): 798–806. doi: 10.1093/infdis/jiz652 31830250 PMC7399704

[8] Hsu CY, Lin CL, Kao CH. Balanitis is a risk factor for herpes zoster. Eur J Clin Microbiol Infect Dis. 2015; 34: 985–990. doi: 10.1007/s10096-015-2314-0 25596845

[9] Hsu CY, Wang YC, Kao CH. Dyshidrosis is a risk factor for herpes zoster. J Eur Acad Dermatol Venereol. 2015; 29: 2177–2183. doi: 10.1111/jdv.13175 25917643

[10] Hsu CY, Ke DS, Lin CL, Kao CH. Risk of herpes zoster in patients with adhesive capsulitis of the shoulder. Int J Environ Res Public Health. 2020; 17(10): 3592. doi: 10.3390/ijerph17103592 32443791 PMC7277430

[11] Hsu CY, Lin CL, Kao CH. Association between chronic interstitial cystitis and herpes zoster. Int J Environ Res Public Health. 2020; 17(7): 2228. doi: 10.3390/ijerph17072228 32224999 PMC7177600

[12] Ke DS, Hsu CY, Lin CL, Hsu CY, Kao CH. Herpes zoster in patients with sciatica. BMC Musculoskelet Disord. 2020; 21(1): 813. doi: 10.1186/s12891-020-03847-5 33278895 PMC7719251

[13] Hsu CY, Ke DS, Lin CL, Kao CH. Association between lateral epicondylitis and the risk of herpes zoster development. Postgrad Med. 2021; 133(1): 96–101. doi: 10.1080/00325481.2020.1816713 32853042

[14] Hsu CY, Ke DS, Lin CL, Kao CH. Risk of herpes zoster infection in men with varicocele. Postgrad Med. 2021 Feb 26: 1–5. Online ahead of print. doi: 10.1080/00325481.2021.1893066 33605831

[15] Liao CH, Chang CS, Muo CH, Kao CH. High prevalence of herpes zoster in patients with depression. J Clin Psychiatry. 2015; 76(9): e1099–1104. doi: 10.4088/JCP.14m09311 26455673

[16] Rapti E, Damigos D, Apostolara P, Roka V, Tzavara C, Lionis C. Patients with chronic pain: evaluating depression and their quality of life in a single center study in Greece. BMC Psychol. 2019; 7(1): 86. doi: 10.1186/s40359-019-0366-0 31864407 PMC6925892

[17] Orhurhu V, Olusunmade M, Akinola Y, Urits I, Orhurhu MS, Viswanath O, et al. Depression trends in patients with chronic pain: an analysis of the nationwide inpatient sample. Pain Physician. 2019; 22(5): e487–e494. 31561661

[18] Morete MC, Solano JPC, Boff MS, Filho WJ, Ashmawi HA. Resilience, depression, and quality of life in elderly individuals with chronic pain followed up in an outpatient clinic in the city of São Paulo, Brazil. J Pain Res. 2018; 11: 2561–2566. doi: 10.2147/JPR.S166625 30464576 PMC6209073

[19] Humo M, Lu H, Yalcin I. The molecular neurobiology of chronic pain-induced depression. Cell Tissue Res. 2019; 377(1): 21–43. doi: 10.1007/s00441-019-03003-z 30778732

[20] Choi HG, Kim EJ, Lee YK, Kim M. The risk of herpes zoster virus infection in patients with depression: a longitudinal follow-up study using a national sample cohort. Medicine (Baltimore). 2019; 98: e17430. doi: 10.1097/MD.0000000000017430 31577760 PMC6783196

[21] Zorrilla EP, Luborsky L, McKay JR, Rosenthal R, Houldin A, Tax A, et al. The relationship of depression and stressors to immunological assays: a meta-analytic review. Brain Behav Immun 2001; 15(3): 199–226. doi: 10.1006/brbi.2000.0597 11566046

[22] Miller AH. Depression and immunity: a role for T cells? Brain Behav Immun 2010; 24(1): 1–8. doi: 10.1016/j.bbi.2009.09.009 19818725 PMC2787959

[23] Irwin M, Costlow C, Williams H, Artin KH, Chan CY, Stinson DL, et al. Cellular immunity to varicella-zoster virus in patients with major depression. J Infect Dis. 1998; 178 Suppl 1: S104–108. doi: 10.1086/514272 9852986

[24] Livengood JM. The role of stress in the development of herpes zoster and postherpetic neuralgia. Curr Rev Pain. 2000; 4(1): 7–11. doi: 10.1007/s11916-000-0003-9 10998709

[25] Dai YX, Yeh FY, Shen YJ, Tai YH, Huang N, Chang YT, et al. Cigarette smoking and risk of herpes zoster: a population-based cohort study in Taiwan. Clin Exp Dermatol. 2021 Mar 24. doi: 10.1111/ced.14650 Online ahead of print. 33763912

