# Supplementary material for: Plantar fascial fibromatosis and herpes zoster
Source: PLoS One. 2021 Nov 24;16(11):e0259942. doi: 10.1371/journal.pone.0259942 (PMC8612523; doi:10.1371/journal.pone.0259942)
Supplement: S1 File — (PDF) [file pone.0259942.s001.pdf]

Wallace Academic Editing

English Editing Certificate

This certifies that the paper **Plantar fascial fibromatosis and herpes zoster** has been edited by Katie Fonseca on July 28, 2020 and is considered to be improved in grammar, punctuation, spelling, verb usage, sentence structure, conciseness, general readability, writing style, and native English usage to the best of the editor's ability.

KF

Best regards,
Wallace Academic Editing

Phone No.: +886-2-2555-5830
Website: http://www.editing.tw
Email: editing@editing.tw
Address: 3F., No.180, Chang'an W. Rd., Datong Dist., Taipei City
